# Symptom Shifting From Nonsuicidal Self-Injury to Substance Use and Borderline Personality Pathology

**DOI:** 10.1001/jamanetworkopen.2024.44192

**Published:** 2024-11-08

**Authors:** Annekatrin Steinhoff, Marialuisa Cavelti, Julian Koenig, Corinna Reichl, Michael Kaess

**Affiliations:** 1 University Hospital of Child and Adolescent Psychiatry and Psychotherapy, University of Bern, Bern, Switzerland; 2 Faculty of Medicine and University Hospital Cologne, Department of Child and Adolescent Psychiatry, Psychosomatics and Psychotherapy, University of Cologne, Cologne, Germany; 3 Department of Child and Adolescent Psychiatry, Center for Psychosocial Medicine, University Hospital Heidelberg, Heidelberg, Germany

## Abstract

**Importance:**

A decline in nonsuicidal self-injury (NSSI) behavior is often viewed as an indication of mental health improvement when treating adolescents and young adults with borderline personality pathology. However, evidence shows that an initial decrease in NSSI behavior does not always signal recovery, and potential symptom shifting needs to be considered.

**Objective:**

To examine the codevelopment of NSSI and substance use in adolescents and young adults receiving treatment for NSSI behavior and to explore whether shifting from NSSI behavior to substance use is associated with emerging or persistent borderline personality pathology.

**Design, Setting, and Participants:**

This cohort study used data from the AtR!Sk study. The study included a consecutive sample of adolescents and young adults with NSSI behavior who presented to a specialized outpatient clinic for early intervention of borderline personality pathology. A baseline assessment (between May 3, 2016, and December 19, 2019) and 2 annual follow-up assessments were conducted. Data were analyzed from April 15, 2023, to September 5, 2024.

**Main Outcomes and Measures:**

The frequencies of NSSI behavior and substance use were self-reported. Diagnostic interviews were carried out to assess borderline personality pathology. Growth mixture models were specified to identify latent classes with different joint trajectories of NSSI behavior and substance use, and the classes were compared for the number of fulfilled borderline personality disorder (BPD) diagnostic criteria.

**Results:**

Overall, 277 adolescents and young adults (249 [89.9%] female; mean [SD] age at baseline, 14.9 [1.5] years) were included in the study (number at first follow-up, 135; number at second follow-up, 82). Three latent classes were extracted from the data. A decline in NSSI behavior following treatment was common. Class 1 (estimated class count: 32.5; 11.7% of participants) was further characterized by a decline in substance use; class 2 (210.1; 75.9% of participants), by a moderate increase in substance use; and class 3 (34.4; 12.4% of participants), by a strong increase in substance use. The number of fulfilled BPD diagnostic criteria in class 1 (mean [SE], 4.64 [0.40]; comparison: χ^2^, 11.64; *P* < .001) and class 3 (mean [SE], 4.29 [0.41]; comparison: χ^2^, 5.98; *P* = .01) was greater than that in class 2 (mean [SE], 3.18 [0.15]) at baseline. The number of fulfilled BPD criteria remained high at the second follow-up assessment in class 3 (mean [SE], 5.15 [0.84]) but not in class 1 (mean [SE], 2.05 [0.54]).

**Conclusions and Relevance:**

In this cohort study of adolescent patients with NSSI behavior, a decline in this behavior was commonly paired with an increase in substance use. This finding suggests that a decrease in NSSI behavior alone may be insufficient to indicate treatment success. Monitoring the joint trajectories of NSSI behavior and substance use may be a promising avenue toward early detection and targeted treatment of adolescent borderline personality pathology.

## Key Points

**Question:**

How common is symptom shifting from nonsuicidal self-injury (NSSI) behavior to substance use among adolescents and young adults with NSSI, and is symptom shifting associated with borderline personality pathology?

**Findings:**

In this cohort study of 277 adolescents and young adults, growth mixture models revealed heterogeneous cotrajectories of NSSI behavior and substance use. A decline in NSSI behavior that was paired with a sharp increase in substance use was associated with the emergence or persistence of a relatively high number of borderline personality disorder symptoms.

**Meaning:**

Findings of this cohort study suggest that, since many adolescents and young adults initially presenting to clinics with NSSI behavior increasingly engage in substance use, a decrease in NSSI behavior alone may be insufficient to indicate treatment success.

## Teaser

This cohort study examines the joint development of substance use and nonsuicidal self-injury (NSSI) behavior to explore whether shifting from NSSI to substance use is associated with emerging or persistent borderline personality disorder among adolescents receiving treatment for NSSI behavior .

## Introduction

Adolescence is a typical onset period of mental illness, and early detection and effective intervention are important to prevent chronic mental disorders and associated health risks in adulthood.^1,2,3^ Borderline personality pathology is associated with severe and prolonged psychiatric and social impairments.^4,5,6^ Borderline personality pathology typically emerges between middle and late adolescence and is characterized by pervasive affective, interpersonal, and identity instability as well as increased impulsivity.^4,5,7,8^ Borderline personality pathology often manifests as self-harm, such as nonsuicidal self-injury (NSSI) behavior—the deliberate infliction of harm upon one’s own body tissue without suicidal intent.^9^ NSSI behavior may prospectively predict borderline personality pathology development,^10^ and a decline in NSSI is often viewed as indication of mental health improvement. Indeed, important randomized clinical trials in the field of adolescent borderline personality pathology have used self-harm, including NSSI behavior, as their primary end point.^11,12^ However, evidence shows that treatment responses are heterogeneous and an (initial) decrease in NSSI behavior does not always signal (full) recovery.^13,14^ One potential reason may be that some adolescent patients successively engage in other unhealthy behaviors, such as substance use.

On average, NSSI behavior and substance use follow different age-graded courses. The prevalence of NSSI behavior onset and past-year engagement in NSSI behavior typically peak between early and middle adolescence,^15,16,17^ although within-person persistence of NSSI behavior and later onsets are common.^18^ By contrast, the prevalence of substance use increases from early adolescence to early adulthood.^19,20^ Given the partly overlapping motives for engaging in these behaviors (eg, affect regulation and alleviation of distress),^21,22^ their differential developmental trends may reflect frequent individual shifts from NSSI behavior to substance use. The literature reports positive correlations between these behaviors,^23,24^ including on the within-person level.^25^ Existing evidence from general population samples suggests that the substitution of deliberate self-harm (ie, NSSI and suicidal behaviors) with risky patterns of substance use across adolescence is associated with borderline personality pathology.^26^ To our knowledge, these associations have not been investigated in clinical adolescent samples.

We examined the following: (1) how NSSI behavior and substance use typically codevelop in adolescents and young adults initially presenting with NSSI behavior and receiving specialized treatment; (2) whether shifting from NSSI behavior to substance use is a common pattern in this patient population; and (3) how different joint trajectories of NSSI behavior and substance use are associated with borderline personality pathology. A main assumption was that adolescent patients show heterogeneous joint trajectories of NSSI behavior and substance use, and that symptom shifting from NSSI behavior to substance use following treatment may be associated with emerging or persistent borderline personality pathology.

## Methods

### Participants and Procedures

This cohort study was carried out in accordance with the Declaration of Helsinki,^27^ and ethics approval was obtained from the ethics committee of the Medical Faculty at the University of Heidelberg, Germany. Written informed consent was obtained from participants and, if they were younger than 16 years of age, their legal guardians. This report follows the Strengthening the Reporting of Observational Studies in Epidemiology (STROBE) guideline.

Participants were recruited from a specialized outpatient clinic, called AtR!Sk, for adolescents with emerging or first presentation of borderline personality disorder (BPD) at the Department of Child and Adolescent Psychiatry, Centre for Psychosocial Medicine at the University of Heidelberg. The clinic provides low-threshold access to initial contact with clinical professionals and evidence-based diagnostics, with the aim of early detection of BPD and intervention.^28^ Following the stepped-care approach, patients are offered tailored intervention considering individual severity of symptoms and treatment response.^28^ Short-term cognitive behavioral therapy^13,29^ is followed by dialectical behavioral therapy for adolescents (DBT-A)^30^ for individuals with persistent symptoms, along with psychiatric management and specialist crisis involvement. Starting in 2013, patients were consecutively invited to participate in the AtR!Sk cohort study. This cohort included patients who received the therapy just described and patients who diverged from this standard procedure by not engaging in proposed interventions, dropping out from treatment early or needing inpatient treatment. Inpatient treatment was less standardized but multimodal and interdisciplinary and was conducted in accordance with the DBT-A principles. In addition, weekly DBT-A–informed individual psychotherapy and skills group were conducted.

At clinic entry, a comprehensive diagnostic assessment was carried out; annual follow-up assessments were conducted thereafter. We used data collected at baseline and 2 follow-up assessments from participants who completed the baseline assessment between 2016 (when risk-taking behaviors were first assessed) and 2019 and reported a minimum of 5 NSSI incidents during the previous year at baseline.

### Variables

NSSI behavior was self-reported at baseline and both follow-up assessments based on the German version of the self-injurious thoughts and behaviors interview.^31,32^ Participants were asked whether they had engaged in NSSI behavior during the previous year and, if yes, how often. Participants who reported that they had not engaged in NSSI behavior during the previous year were coded 0 for NSSI behavior frequency. To compress the distribution and compare relative rather than absolute frequencies, we included a log-transformed version of this variable in the statistical models (log [NSSI behavior +1]).

Substance use was self-reported at baseline and both follow-up assessments as part of a semistructured interview and question formats similar to those in the Youth Risk Behavior Survey.^33^ The participants reported how often during the previous year they had used alcohol or illicit substances (1, never; 2, sometimes; 3, at least once per month; 4, at least once per week; 5, 2 to 3 days per week; 6, almost daily; 7, daily). We computed a sum score to integrate information on the overall frequency of any substance use and its severity in terms of polysubstance use (eg, a score of 14 represented daily use of alcohol and other substances).

Borderline personality pathology was assessed using the German version of the Structured Clinical Interview for *Diagnostic and Statistical Manual of Mental Disorders* (Fourth Edition) Axis II disorders,^34^ which has been validated for use with adolescent samples.^35^ We used the number of fulfilled BPD criteria to indicate severity of borderline personality pathology at baseline and at the second follow-up assessment. For sociodemographic characteristics, we included participants’ age and sex (dummy coded, 0 = male, 1 = female) assessed at baseline.

### Statistical Analysis

To examine the mean joint trajectories of log-transformed NSSI behavior and substance use, we specified a latent growth model with parallel processes and linear slopes. We estimated regression paths from intercepts (ie, baseline levels) to slopes (ie, subsequent change), adjusted for age and sex. Model fit was evaluated using the χ^2^ test (nonsignificant result indicated adequate fit), the comparative fit index (values above 0.95 indicated adequate fit), and the root mean square error of approximation (values below 0.08 reflected adequate fit).^36,37^

To identify heterogenous groups with different joint trajectories of NSSI behavior and substance use, we specified growth mixture models in a latent variable framework.^38^ To identify the optimal number of classes, we compared models with 1 to 4 latent classes based on the Akaike information criterion and the bayesian information criterion, with lower values indicating better model fit; entropy, indicating classification precision (values closer to 1 indicating more precision); and conceptual interpretability.^39,40,41,42^ In addition, likelihood-based tests^43^ compare a solution with *k* classes to one with *k* − 1 classes and provide a *P* value indicating whether an additional class improved the model. To facilitate the estimation of 3- and 4-class solutions, we constrained an otherwise negative but small and nonsignificant residual variance of the latent slope factor of substance use (variance = −0.11, SE = 0.86, *P* = .90) to zero. Associations of age and sex with class were assessed with logistic regression odds ratios (ORs) and 95% CIs. Between-class differences in borderline personality pathology at baseline and the second follow-up assessment were tested using the Bolck, Croon, and Hagenaars method, which provides mean score comparisons.^44,45^

Descriptive analyses were conducted using SPSS, version 25 (SPSS Inc); growth models were estimated using Mplus, version 8 (Muthén and Muthén; output files are provided in in an online repository^46^).^47^ We used maximum likelihood estimation with robust standard errors and full information maximum likelihood to handle missing data. This procedure facilitates the inclusion of all available data, thereby reducing potential bias due to selective attrition.^48^

Analyses were conducted from April 15, 2023, to September 5, 2024. A 2-sided *P* < .05 was considered significant.

## Results

At baseline, the ages of 277 participants ranged from 12 to 19 years (mean [SD], 14.9 [1.5] years), and most participants were female (249 [89.9%]; 28 [10.1%] male) (Table 1). One-third of participants attended schools requiring either high (99 [35.7%]) or medium (94 [33.9%]) achievement levels. Parental separation was a common experience (141 participants [50.9%]). Overall, 135 participants completed the first follow-up assessment a mean (SD) of 12.39 (1.63) months after baseline, and 82 participants completed the second follow-up assessment a mean (SD) of 11.24 (2.32) months after the first follow-up. At least 2 assessments were completed by 150 participants, including 67 who completed all 3 assessments. Individuals who participated in at least 1 follow-up assessment reported less substance use (Cohen *d,* −0.34 [95% CI, −0.58 to −0.10]; *P* = .006) and more log-transformed NSSI behavior (Cohen *d*, 0.30 [95% CI, 0.07-0.54]; *P* = .01) than individuals who did not participate in the first or second follow-up assessment.

**Table 1.** Sample Characteristics and Descriptive Statistics for Study Variables of 277 Included Participants

Variable	Participants
Age, baseline, mean (SD), y	14.9 (1.5)
Sex, baseline, No. (%)	
Female	249 (89.9)
Male	28 (10.1)
School type: baseline, No. (%)	
Low achievement level (Hauptschule)	31 (11.2)
Medium achievement level (Realschule)	94 (33.9)
High achievement level (Gymnasium)	99 (35.7)
Other	53 (19.1)
Parental relationship status: baseline, No. (%)	
Living together	121 (43.7)
Separated	141 (50.9)
Death of at least 1 parent or unknown status	15 (5.5)
Nonsuicidal self-injury behavior: past-year frequency, mean (SD)	
Baseline^a^	63.78 (70.18)
1-y Follow-up^b^	47.56 (68.43)
2-y Follow-up^c^	18.20 (30.70)
Substance use: sum score, frequency of use for different substances, mean (SD)	
Baseline	4.14 (2.42)
1-y Follow-up	4.31 (2.25)
2-y Follow-up	4.43 (2.08)
Borderline personality disorder, mean (SD), No. of criteria fulfilled	
Baseline	3.49 (2.17)
2-y Follow-up	2.76 (2.30)

^a^ Range, 5 to 350; median (IQR), 40 (66).

^b^ Range, 0 to 335; median (IQR), 15 (57).

^c^ Range, 0 to 145, median (IQR), 4 (21).

The overall latent growth model showed adequate fit to the data (χ^2^_12_, 15.68, *P* = .21; comparative fit index, 0.97; and root mean square error of approximation, 0.03). On average, NSSI behavior declined over time (mean [SE] latent intercept, 3.63 [0.06]; *P* < .001; linear slope [SE], −0.95 [0.08]; *P* < .001), and substance use increased over time (intercept [SE], 4.16 [0.15]; *P* < .001; slope [SE], 0.27 [0.11]; *P* = .02). A higher intercept of NSSI behavior was associated with a steeper subsequent decline of NSSI behavior (*b* = −0.35; SE = 0.11; *P* = .002). The intercept and slope of substance use were not associated (*b* = −0.17; SE = 0.16; *P* = .30), and no cross-concept associations between intercepts and slopes emerged (*b* = −0.05; SE = 0.05; *P* = .33) for the regression of NSSI behavior slope on substance use intercept (*b* = −0.10; SE = 0.12; *P* = .38 for substance use slope on NSSI intercept). Older age was associated with a higher substance use intercept (*b* = 0.54; SE = 0.08; *P* < .001).

Model fit indicators for the growth mixture models (Table 2) supported the selection of the 3-class solution. The Akaike information criterion decreased with an increase in the number of classes, at a decelerating rate. The bayesian information criterion reached a minimum when extracting 3 classes. Likelihood-based testing with bootstrapping suggested a 3-class rather than 2-class solution when we additionally computed the bootstrapped likelihood ratio test for the solution with the lowest bayesian information criterion, as recommended based on simulation studies.^43,49^ The test indicated that the 3-class solution fit better than the 2-class solution (2 times the log of the likelihood difference, 78.09; difference in number of parameters, 7; *P* < .001). Entropy for the 3-class solution indicated high classification precision and, from a conceptual point of view, 3 well-defined classes were extracted.

**Table 2.** Indicators of Model Fit, Entropy, and Class Sizes From Growth Mixture Models With Different Numbers of Latent Classes

Model, No. of classes	AIC	BIC	VLMR: 2 × log likelihood difference (difference in No. of parameters)	*P* value from VLMR		Entropy	Class sizes, %
1	3753.49	3851.33	NA	NA		NA	NA
2	3640.17	3763.38	127.3 (7)	<.001		0.96	85.7, 14.3
3^a^	3600.60	3741.94	49.9 (7)	.13		0.89	11.7, 75.9, 12.4
4	3588.45	3755.16	26.2 (7)	.46		0.68	44.5, 31.5, 11.7, 12.4

Abbreviations: AIC, Akaike information criterion; BIC, bayesian information criterion; NA, not applicable; VLMR, Vuong-Lo-Mendell-Rubin.

^a^ The solution selected for further analyses.

The Figure shows the class-specific joint trajectories of NSSI behavior and substance use; Table 3 provides class-specific means of the latent growth factors. Participants in class 3 reported lower initial levels of NSSI behavior than others; a subsequent decline in NSSI behavior was common across all classes. Participants in class 1 reported the highest initial levels of substance use, followed by participants in classes 3 and 2. While participants in class 1 showed a significant subsequent decline in substance use, participants in other classes reported increasing substance use. Substance use increased especially strongly in class 3. We termed class 1 (estimated class count, 32.5 participants [11.7%]) *joint decline of NSSI behavior and substance use*; class 2 (210.1 participants [75.9%]), *decline of NSSI behavior, increase of substance use at moderate level and pace*; and class 3 (34.4 participants [12.4%]), *decline of NSSI behavior, increase of substance use at high level and pace*.

**Figure.**
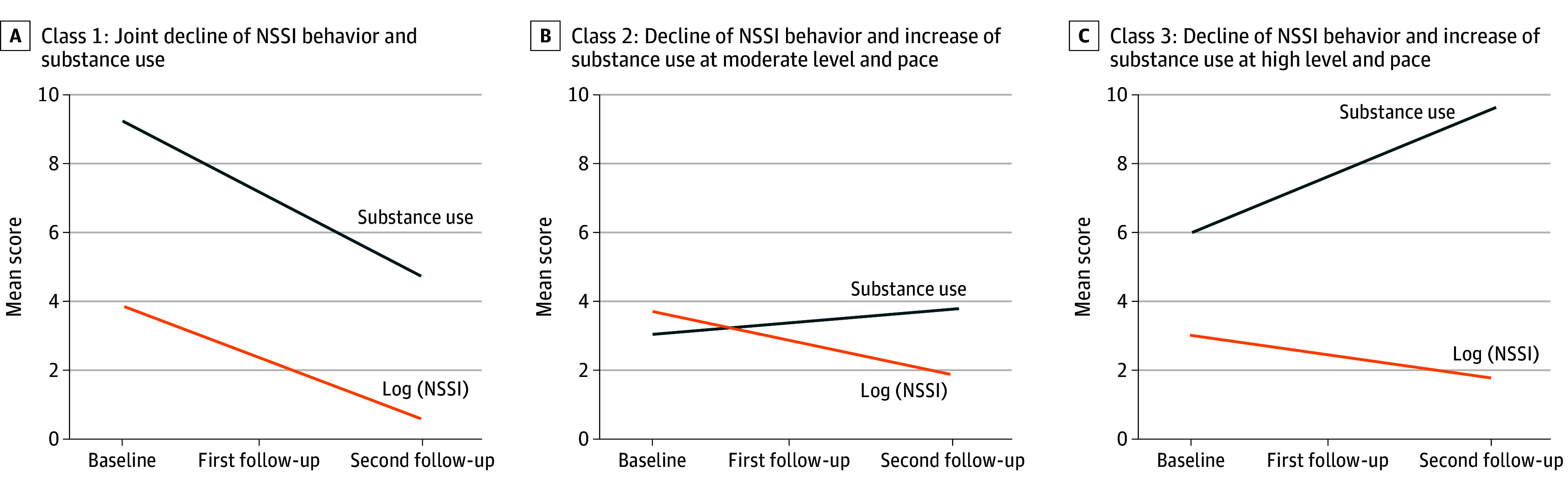
Joint Trajectories of Log-Transformed Nonsuicidal Self-Injury (NSSI) Behavior and Substance Use in 3 Latent Classes

**Table 3.** Growth Parameters for 3 Latent Classes

Variable	Class 1: joint decline of NSSI behavior and substance use values		Class 2: decline of NSSI behavior and increase of substance use values at moderate level and pace		Class 3: decline of NSSI behavior and increase of substance use values at high level and pace	
	Estimated mean (SE)	*P* value	Estimated mean (SE)	*P* value	Estimated mean (SE)	*P* value
Participants, No. (%)	32.5 (11.7)	NA	210.1 (75.9)	NA	34.4 (12.4)	NA
Log NSSI behavior latent intercept	3.84 (0.23)	<.001	3.70 (0.07)	<.001	3.02 (0.21)	<001
Log NSSI behavior latent slope	−1.62 (0.23)	<.001	−0.91 (0.09)	<.001	−0.62 (0.24)	.009
Substance use latent intercept	9.23 (0.38)	<.001	3.07 (0.09)	<.001	6.06 (0.49)	<001
Substance use latent slope	−2.25 (0.42)	<.001	0.37 (0.08)	<.001	1.79 (0.37)	<.001

Abbreviations: NA, not applicable; NSSI, nonsuicidal self-injury.

At baseline, class 1 (fulfilled borderline personality pathology criteria mean [SE], 4.64 [0.40]) and class 3 (fulfilled borderline personality pathology criteria mean [SE], 4.29 [0.41]) were characterized by higher levels of borderline personality pathology than class 2 (fulfilled borderline personality pathology criteria mean [SE], 3.18 [0.15]) (Table 4). At the second follow-up assessment, fulfilled borderline personality pathology criteria in class 1 (mean [SE], 2.05 [0.54]) had declined to that of class 2 (mean [SE], 2.53 [0.29]), which remained relatively low; class 3 had the highest number of fulfilled borderline personality pathology criteria (mean [SE], 5.15 [0.84]). These results suggest that the joint decrease in NSSI behavior and substance use in class 1 was a behavioral manifestation of an underlying decline in borderline personality pathology, whereas adolescents and young adults in class 3 experienced symptom shifting from NSSI behavior to substance use—a manifestation of emerging or persistent borderline personality pathology.

**Table 4.** Number of Fulfilled BPD Criteria in 3 Latent Classes and Comparisons Between Classes

Assessment	No. of fulfilled BPD criteria, mean (SE)			Class 1 vs class 2		Class 1 vs class 3		Class 2 vs class 3	
	Class 1	Class 2	Class 3	χ^2^	*P* value	χ^2^	*P* value	χ^2^	*P* value
Baseline	4.64 (0.40)	3.18 (0.15)	4.29 (0.41)	11.64	.001	0.33	.57	5.98	.01
Second follow-up	2.05 (0.54)	2.53 (0.29)	5.15 (0.84)	0.64	.42	9.00	.003	8.34	.004

Abbreviation: BPD, borderline personality disorder.

Regarding sociodemographic characteristics, older age was associated with higher odds of being in classes 1 (OR, 1.32 [95% CI, 1.06-1.65]) and 3 (OR, 1.44 [95% CI, 1.08-1.92]) vs class 2. Female sex was associated with lower odds of being in class 1 vs 2 (OR, 0.31 [95% CI, 0.12-0.82]).

In sensitivity analyses, we compared the 3 classes regarding borderline personality pathology while excluding either BPD criterion 4 (impulsive behaviors) or criterion 5 (suicidal and NSSI behaviors) (eTables 1 and 2 in Supplement 1).^46^ The results replicated those from the main analyses, except for the difference between classes 2 and 3 at baseline when excluding criterion 4.

## Discussion

The results from this cohort study showed that, on average, a decline in NSSI behaviors following specialized treatment^13^ codeveloped with an increase in substance use among adolescents and young adults receiving psychiatric care. To some extent, this finding mirrors the typical age-related increase in substance use across adolescence in the general population.^19^ However, our study also indicated that receiving (effective) treatment for NSSI behaviors was not necessarily also associated with a reduction in risk-taking behaviors, such as substance use. Since increasing substance use can pose severe health risks for adolescents^50,51^ and may signal symptom shifting associated with emerging or persistent borderline personality pathology, decreasing levels of NSSI behavior alone are insufficient to indicate therapy success. Our findings provide a suggestion for how the monitoring of joint developmental patterns of NSSI behavior and substance use in adolescents and young adults receiving psychiatric care can inform prevention and targeted treatment of borderline personality pathology.

Two groups of equal size (ie, classes 1 and 3, including 11.7% and 12.4% of participants, respectively) were characterized by relatively extreme trajectories of substance use. In line with previous evidence,^52^ the combination of high initial levels of substance use and NSSI behavior in these classes was associated with relatively high initial levels of borderline personality pathology. As substance use and borderline personality pathology typically emerge and increase in middle to late adolescence,^7,20^ the participants’ older age, compared with class 2, may partly explain these patterns.

The joint decline in NSSI behavior and substance use among patients in class 1 may indicate a general disengagement from maladaptive strategies for emotion regulation.^21,22^ Indeed, the low level of borderline personality pathology at the second follow-up for adolescents and young adults in class 1, which contrasts with their high initial borderline personality pathology level, suggests an overall improvement of mental health following treatment. The characterization of class 3 indicates that, among adolescents and young adults who initially present to clinics with NSSI behavior, a subsequent sharp increase in substance use can signal emerging or persistent borderline personality pathology, even if NSSI declines. Here, our study adds to previous findings, based on a general population sample, indicating that borderline personality pathology is associated with shifting from NSSI behavior to risky substance use.^26^ A reason may be that substance use can serve similar functions as NSSI behavior related to emotion dysregulation.^24^ At the same time, patients may perceive their increasing substance use as less problematic than NSSI behavior, given the normative age-related increase in substance use in the general adolescent population^53^ and thus, likely among many of their healthy peers.^54^

Previous research has shown that the concurrent or subsequent co-occurrence of self-harm and risk-taking behaviors, and specifically NSSI behavior and substance use, is associated with severe mental health and social impairments across adolescence and thereafter.^55,56^ Our new findings suggest that borderline personality pathology may be a common underlying factor, as borderline personality pathology is generally associated with severe psychosocial impairments.^8^ To avoid the risk of therapy failure, it may be advisable to monitor patients’ trajectories of substance use in addition to NSSI behavior during therapy and afterwards (including, for example, by advising patients how to self-monitor their behavioral trajectories) and provide timely and regular screenings for borderline personality pathology if a patient seems to engage increasingly in other maladaptive strategies for self-regulation. However, it may not always be easy to promptly differentiate between normative and problematic increases in substance use among patients with NSSI behavior. Our findings regarding class 3 provide 2 helpful indicators. First, an increase in substance use should be considered a red flag, especially if emerging borderline personality pathology has already been detected. Second, class 3 reached a mean level of substance use that indicates polysubstance use (ie, participants must have reported alcohol *and* illicit substances use at least “sometimes”). Other research has shown that polysubstance use is especially harmful,^57^ and our characterization of class 3 may be taken as preliminary evidence that there is an association with borderline personality pathology.

To better understand whether cotrajectories of NSSI behavior and substance use as observed here are, indeed, best conceived of as symptom shifting, a promising next step could be qualitative research asking adolescents with lived experience about their own perceptions of their behavioral trajectories and related reasoning. Research considering dimensional conceptualizations of personality disorders^58,59^ may explore the specific roles of impaired self-functioning and interpersonal functioning in the cotrajectories of NSSI behavior and substance use. Furthermore, the specificity of certain cotrajectories to borderline personality pathology vs other mental illnesses, such as mood disorders, needs to be investigated. While our aim was to provide first insights into cotrajectories between NSSI behavior and overall substance use, future studies should explore whether our findings generalize across substances (eg, investigate alcohol and illicit drug use separately). Cotrajectories including additional self-harm and risk-taking behaviors (eg, pathological media use, disordered eating, suicide attempts) also need to be considered. Finally, to better understand mechanisms underlying symptom shifting, research may examine the role of NSSI thoughts and urges.

### Strengths and Limitations

The strengths of our study include the prospective longitudinal design and advanced statistical modeling revealing well-differentiated joint trajectories of NSSI behavior and substance use. Another strength is the use of a well-established diagnostic tool to assess borderline personality pathology according to common clinical practice.

Limitations include, first, that NSSI behavior and substance use were assessed using self-reports. These may underestimate the frequency of the behaviors due to social desirability or recall bias.^60^ However, underreporting would primarily impair the estimation of overall levels, rather than developmental patterns. Second, our sample included predominantly female participants, and future research with more balanced samples is needed to investigate whether our findings generalize to other genders. Third, time intervals between assessments were not the same for all participants, which may affect the results (eg, underestimation of the number of trajectory groups). Finally, there was some indication of selective missingness, and the latter could also be associated with unobserved variables relevant to treatment success. Although we used state-of-the-art techniques to include all available data in our models, future research, ideally with larger samples and higher retention rates, is needed to replicate our findings.

## Conclusions

In this cohort study of adolescent patients initially presenting with NSSI behavior, shifting from NSSI behavior to substance use was common and appeared to be associated with emerging or persistent borderline personality pathology. Observing a patient’s trajectory of NSSI behavior alone may be insufficient to evaluate treatment success. Monitoring the joint trajectories of NSSI behavior and substance use may be a more promising avenue toward timely, well-targeted treatment with the aim to prevent chronic psychiatric impairments.

## Notes

Supplement 1. **eTable 1.** Number of Fulfilled BPD Criteria, Excluding Criterion 4, in 3 Latent Classes and Comparisons Between Classes (χ^2^ tests) **eTable 2.** Number of Fulfilled BPD Criteria, Excluding Criterion 5, in 3 Latent Classes and Comparisons Between Classes (χ^2^ tests)

Supplement 2.
Data Sharing Statement
