# Supplementary material for: Symptom Shifting From Nonsuicidal Self-Injury to Substance Use and Borderline Personality Pathology
Source: JAMA Netw Open. 2024 Nov 8;7(11):e2444192. doi: 10.1001/jamanetworkopen.2024.44192 (PMC11549661; doi:10.1001/jamanetworkopen.2024.44192)
Supplement: Supplement 1. — eTable 1. Number of Fulfilled BPD Criteria, Excluding Criterion 4, in 3 Latent Classes and Comparisons Between Classes (χ2 tests) eTable 2. Number of Fulfilled BPD Criteria, Excluding Criterion 5, in 3 Latent Classes and Comparisons Between Classes (χ2 tests) [file jamanetwopen-e2444192-s001.pdf]

Supplementary Online Content

Steinhoff A, Cavelti M, Koenig J, Reichl C, Kaess M. Symptom shifting from nonsuicidal self-injury to substance use and associations with borderline personality pathology. *JAMA Netw Open*. 2024;7(11):e2444192. doi:10.1001/jamanetworkopen.2024.44192

eTable 1. Number of Fulfilled BPD Criteria, Excluding Criterion 4, in 3 Latent Classes and Comparisons Between Classes (Chi-square tests)

eTable 2. Number of Fulfilled BPD Criteria, Excluding Criterion 5, in 3 Latent Classes and Comparisons Between Classes (Chi-square tests)

This supplementary material has been provided by the authors to give readers additional information about their work.

© 2024 Steinhoff A et al. *JAMA Network Open*.

eTable 1. Number of Fulfilled BPD Criteria, Excluding Criterion 4, in 3 Latent Classes and Comparisons Between Classes (Chi-square tests)

Assessment	**No. of fulfilled BPD criteria, mean (SE)**			**Class 1 vs. class 2**		**Class 1 vs. class 3**		**Class 2 vs. class 3**	
	**Class 1**	**Class 2**	**Class 3**	X^2	*p*	X^2	*p*	X^2	*p*
Baseline	3.86 (0.36)	3.05 (0.14)	3.77 (0.38)	4.43	0.04	0.02	0.88	3.04	0.08
Second follow-up	1.97 (0.57)	2.34 (0.26)	4.11 (0.71)	0.35	0.55	4.93	0.03	5.07	0.02

© 2024 Steinhoff A et al. *JAMA Network Open*.

eTable 2. Number of Fulfilled BPD Criteria, Excluding Criterion 5, in 3 Latent Classes and Comparisons Between Classes (Chi-square tests)

Assessment	**No. of fulfilled BPD criteria, mean (SE)**			**Class 1 vs. class 2**		**Class 1 vs. class 3**		**Class 2 vs. class 3**	
	**Class 1**	**Class 2**	**Class 3**	X^2	*p*	X^2	*p*	X^2	*p*
Baseline	3.76 (0.37)	2.39 (0.14)	3.43 (0.39)	12.07	0.001	0.32	0.57	5.88	0.02
Second follow-up	1.76 (0.61)	2.04 (0.26)	4.33 (0.87)	0.17	0.68	5.23	0.02	6.07	0.01

Abbreviation. BPD = Borderline Personality Disorder.

© 2024 Steinhoff A et al. *JAMA Network Open*.
