# Supplementary material for: Symptom Shifting From Nonsuicidal Self-Injury to Substance Use and Borderline Personality Pathology
Source: JAMA Netw Open. 2024 Nov 8;7(11):e2444192. doi: 10.1001/jamanetworkopen.2024.44192 (PMC11549661; doi:10.1001/jamanetworkopen.2024.44192)
Supplement: Supplement 2. — Data Sharing Statement [file jamanetwopen-e2444192-s002.pdf]

Data Sharing Statement

Steinhoff. Symptom Shifting from Nonsuicidal Self-Injury to Substance Use and Associations With Borderline Personality Pathology. *JAMA Netw Open*. Published November 08, 2024. doi:10.1001/jamanetworkopen.2024.44192

Data
Data available: Yes
Data types: Deidentified participant data
How to access data: michael.kaess@upd.ch
When available: With publication

Supporting Documents
Document types: None

Additional Information
Who can access the data: researchers whose proposed use of the data has been approved
Types of analyses: for any purpose that aligns with common ethical standards
Mechanisms of data availability: after approval of a proposal, with a signed data access agreement
